# Side effects of working from home: the dangers affecting the well-being of employees: response

**DOI:** 10.47626/1679-4435-2024-11781

**Published:** 2024-08-05

**Authors:** Alberto José Niituma Ogata

**Affiliations:** 1 Centro de Estudos em Planejamento e Gestão de Saúde (FGVsaúde), Escola de Administração de São Paulo, Fundação Getúlio Vargas, São Paulo, SP, Brazil

Dear Editors-in-Chief,

Regarding the article published in this Journal in 2022, titled “Impact on health and well-being of working at home during the SARS-CoV-2 pandemic,”^1^ I would like to comment that the online survey was conducted during a period in which many employees were confined to their homes.

Our questionnaire was adapted from a questionnaire developed by the Institute for Employment Studies (IES) in the United Kingdom, titled “Working at Home Wellbeing Survey,”^2^ which aimed to assess the well-being of individuals who began working from home during the SARS-CoV-2 pandemic.

We also used the World Health Organization-5 Well-Being Index (WHO-5), a well-being assessment tool consisting of five questions with responses rated on a Likert scale in Portuguese. According to this instrument, scores below 13 indicate a low level of well-being.^3^

The methodology involved conducting a cross-sectional descriptive study with a convenience sample of employees working from home and administering the WHO-5 questionnaire and a symptom inventory via an online platform.

The study revealed that low levels of well-being were associated with several physical symptoms, such as lower back, neck, and muscle pains, migraines, and gastrointestinal disorders. This underscores the importance of delving into issues related to the quality of life of employees beyond the pharmacological treatment of symptoms.

The same instrument (WHO-5) was used in a survey conducted by our team at the end of the pandemic, which involved a group of Brazilian employees. The results were published in the Journal of Occupational Environmental Medicine in 2023.^4^
